# Anorexia nervosa-specific home treatment in children and adolescents and their families (the HoT study): a study protocol of a randomized, controlled, multicenter, open-label, parallel group superiority trial

**DOI:** 10.1186/s13063-024-08566-z

**Published:** 2024-11-13

**Authors:** Brigitte Dahmen, Ingar Zielinski-Gussen, Manuel Föcker, Freia Hahn, Tanja Legenbauer, Ulf Thiemann, Astrid Dempfle, Beate Herpertz-Dahlmann

**Affiliations:** 1 Department of Child and Adolescent Psychiatry, Psychosomatics and Psychotherapy, University Hospital, RWTH Aachen University, Neuenhofer Weg 21, 52074 Aachen, Germany; 2 Department of Child and Adolescent Psychiatry, University Hospital Münster, Schmeddingstraße 50, 48149 Münster, Germany; 3 Department of Child and Adolescent Psychiatry, Psychosomatics and Psychotherapy, LVR Clinic Viersen, Horionstraße 14, 41749 Viersen, Germany; 4 LWL University Hospital Hamm for Child and Adolescent Psychiatry, Psychotherapy and Psychosomatic, Ruhr-University Bochum, Heithofer Allee 64, 59071 Hamm, Germany; 5 Department of Child and Adolescent Psychiatry, Psychosomatics and Psychotherapy, LVR Hospital Bonn, Kaiser- Karl-Ring 20, 53111 Bonn, Germany; 6 Institute of Medical Informatics and Statistics, Kiel University and University Hospital Schleswig-Holstein, Brunswiker Str. 10, 24105 Kiel, Germany

**Keywords:** Anorexia nervosa, Home treatment, Stepped-care, Adolescents, Health services research

## Abstract

**Background:**

New treatment approaches are urgently needed to improve the prognosis of children and adolescents with anorexia nervosa (AN). Recently, the feasibility of multidisciplinary home treatment that strongly involves the patients’ parents/caregivers has been investigated. However, no RCT has yet been performed to test the efficacy and safety of this approach compared to standard treatment approaches, such as inpatient treatment.

**Methods:**

In this multicenter randomized-controlled trial, home treatment for children and adolescents with AN aged 12 to 18 years is established at 5 major treatment centers for AN in Germany. Approximately 240 patients who are admitted to the hospital for AN will be included in the trial. After a short inpatient somatic stabilization phase (5–8 weeks), patients are randomized to receive either treatment as usual (TAU), in the form of continued inpatient or day patient treatment, or the newly developed home treatment (HoT) (*n* = 82/arm, *n* = 164 in total). There are three assessments throughout treatment (admission, randomization, and discharge), as well as follow-up assessments at 9 and 12 months after admission. The BMI at 12 months after admission (primary outcome) is compared between groups (adjusted for premorbid BMI and admission BMI); secondary outcomes include eating disorder and general psychopathology, the number and duration of psychiatric rehospitalizations, quality of life, motivation for treatment and treatment satisfaction. Other secondary outcomes include the primary caregivers’ burden and skills in handling the child’s illness and direct treatment costs. Statistical analysis will be based on intention-to-treat principles, using mixed models for repeated measures. (Serious) adverse events are assessed throughout treatment. In addition, the feasibility and implementation of HoT as well as the satisfaction and workload of the members of the multidisciplinary treatment teams in both arms will be assessed.

**Discussion:**

In the case of a positive evaluation, HoT can be considered an effective treatment method to replace or complete established treatment methods, such as IP, for treating AN in children and adolescents. The home treatment setting might shorten inpatient stays in this patient group, increase treatment satisfaction, and help to reduce the risk of rehospitalization, which is associated with a better outcome in this vulnerable patient group.

**Trial registration:**

The trial was registered with the German Clinical Trial Register (DRKS) under the ID DRKS00025925 on November 26, 2021 (prospectively registered): https://drks.de/search/de/trial/DRKS00025925.

## Introduction

### Background and rationale

The World Health Organization considers anorexia nervosa (AN) to be one of the four main disorders associated with “lifelong consequences” in the field of child and adolescent psychiatry [1]. The main characteristics of AN are a significantly low body weight, an immense fear of gaining weight, and an excessive influence of body weight on self-worth. Patients with AN also commonly suffer from additional psychiatric [2] and somatic disorders [3]. A significantly higher mortality rate has been reported due to increased suicidality [4] and (mostly cardiovascular) complications due to long periods of starvation [5]. A recent meta-analysis demonstrated that only approximately 30% of patients with AN fully recover [6]. A shorter duration of untreated illness, especially in adolescence, is related to a greater likelihood of remission [7].

Outpatient treatment (OP) is considered the first-line treatment in children and adolescents with AN [8, 9]. While OP is recommended as the first choice of treatment for adolescents with AN, approximately half of these patients require more intensive care, generally inpatient treatment (IP) [10]. Although IP is often effective in achieving weight gain [11, 12], it is not always sufficiently effective in modifying disordered eating behavior and other symptoms of AN [13]. In addition, long hospitalizations impede the age-appropriate social development of adolescents with AN [14]. On the other hand, shorter IP approaches with primarily nutritional and somatic stabilization are often still associated with high levels of stress and symptoms of depression and anxiety upon discharge from the hospital [15], limiting the potential impact of simply shortening IP. Generally, after discharge from IP, almost all patients with AN initially struggle to maintain a healthy weight, and many patients relapse into disordered eating behaviors such as food restriction and excessive exercising, followed by another round of weight loss [16]. Approximately one third of adolescents with AN who undergo at least one inpatient treatment require rehospitalization within 1 year after discharge [17, 18]. In our own randomized-controlled trial (RCT) on day patient treatment, approximately 30% of patients required a second admission to the hospital during a 2½-year follow-up [19]. In addition, inpatient treatment is associated with the highest treatment costs compared to other treatment modalities [20]. Given the very high readmission rates after IP [21, 22] and increased hospital admissions of children and adolescents with AN—especially during the COVID-19 pandemic [23, 24]—innovative ideas are desperately needed.

The concept of “stepped-care” to facilitate the transition from more intensive (e.g., IP) to less intensive (e.g., day patient (DP) or partial IP) care to shorten hospital stays and to ease the transition between treatment settings has been proposed [25, 26]. Shorter hospitalizations followed by longer, more intensive OP or DP treatments have been at least similarly as effective (and less expensive) as IP alone [27, 28]. Therapeutic approaches that allow young patient to spend more time out of the hospital while still receiving intensive multidisciplinary treatment emphasize the important role of parents/primary caregivers as “co-therapists” whom are relevant to outcomes, especially for children and adolescents with AN [29]. In addition, AN is associated with the development of dysfunctional habits [30], which are mostly exacerbated in the home environment and should be treated where they arise. Thus, approaches to treating adolescent patients with AN in their home environment and involving their families have recently been hypothesized to prevent (re-)hospitalization with promising results in pilot studies [31–33].

In the Aachen model, stepped-care home treatment (HoT) is delivered by a multidisciplinary team of medical doctors, psychotherapists, nurses, nutritionists, and occupational, psychomotor and social therapists for adolescent patients with moderate to severe AN after short-term IP treatment to reduce the IP duration and prevent relapse [31]. In a pilot trial design during the home treatment period itself and at the 12-month follow-up, the patients achieved their target weights, showed significant improvement in ED and general psychopathology, and exhibited strong improvement in motivation to overcome their eating disorder [31, 34]. Additionally, the parents’ burden decreased, and their skills to handle their child with an AN increased throughout HoT [35]. Although these pilot data suggest that HoT is an effective and urgently needed therapeutic program, randomized controlled trials are lacking as a prerequisite for implementing home treatment in general medical care in Germany and elsewhere.

### Objectives

Thus, the current study aimed to close this research gap. This paper describes the protocol for a multicenter randomized-controlled trial with the following research questions:

1. Is the stepped-care home treatment approach “HoT” superior to treatment as usual (TAU) when applied as continuous IP or stepped-care IP/DP in improving body mass index (BMI) at 12 months after hospital admission (primary outcome), AN, and general psychopathology; avoiding rehospitalization, and increasing treatment motivation, quality of life, satisfaction with treatment of both patients and caregivers (secondary outcomes)? Is patient safety ensured during HoT?
2. Is HoT less expensive than TAU in terms of direct costs during differential treatment, i.e., between randomization and discharge?
3. How feasible is HoT, and how do the staff of child and adolescent psychiatric hospitals accept the treatment method of HoT (visiting the patient and his or her family at home) as a precondition for implementing HoT in common departments for child and adolescent psychiatry? How do caregivers’ burden and skills change during HoT?
4. Can this new stepped-care home treatment approach “HoT” be applied to the broad range of young patients with AN who are admitted to hospitals in Germany? How similar are German AN patients in general to the patients in the RCT, and how many could be expected to be suitable for HoT? Can this treatment modality potentially be of widespread importance/be applied to a relevant proportion of treatment-requiring AN patients?

### Trial design

This report of the study protocol and the trial follows the SPIRIT guidelines [36] with a populated SPIRIT checklist. The current project consists of four subprojects to achieve the overall study objectives: (1) a two-arm, multicentre, open-label, parallel group superiority RCT comparing IP or IP/DP (TAU) with IP/HoT in a stepped-care manner for adolescent patients (12–18 years) with severe AN for whom hospital admission was prescribed, (2) a cost analysis of the direct treatment costs, (3) a process evaluation of the feasibility and implementation of the HoT procedure at the participating clinical centers/sites by an analysis of the staff’s appraisal of HoT compared to TAU, and (4) an analysis of the transferability and external validity by comparing the patient data of this sample with a representative German-wide sample.

## Methods: participants, interventions, and outcomes

### Study setting

The trial is located at the coordinating center, the Department of Child and Adolescent Psychiatry, Psychosomatics and Psychotherapy, University Hospital RWTH Aachen (consortium head/PI: Prof. Beate Herpertz-Dahlmann, MD, PhD and Brigitte Dahmen, MD) and the four participating centers, all located in the region of North-Rhine-Westphalia, Germany: The Department of Child and Adolescent Psychiatry and Psychotherapy, University Hospital Münster (PI: PD Manuel Föcker, MD), the LWL University Hospital Hamm for Child and Adolescent Psychiatry, Psychotherapy and Psychosomatics, Ruhr-University Bochum (PI: Prof. Tanja Legenbauer, PhD), the Department of Child and Adolescent Psychiatry, Psychosomatics and Psychotherapy, LVR Clinic Viersen (PI: Freia Hahn, MD), and the Department for Child and Adolescent Psychiatry, LVR Hospital Bonn (PI: Ulf Thiemann, MD). Only hospitals with a specialized ED unit for children and adolescents were included. The data management and statistical planning and analyses are performed by the Institute of Medical Information and Statistics (IMIS) at Kiel University (CAU Kiel), Germany (PI: Prof. Astrid Dempfle, PhD). In addition to the clinical centers, the following German health insurance companies are involved in this collaborative effort: Allgemeine Ortskrankenkasse (AOK) Hamburg-North-Rhine-Westphalia, Deutsche Angestelltenkrankenkasse (DAK), Innungs-Krankenkasse classic (IKK-classic), and Techniker Krankenkasse (TK). All German public health insurance companies agreed to cover the costs of the HoT treatment within this trial.

### RCT

**Fig. 1:**
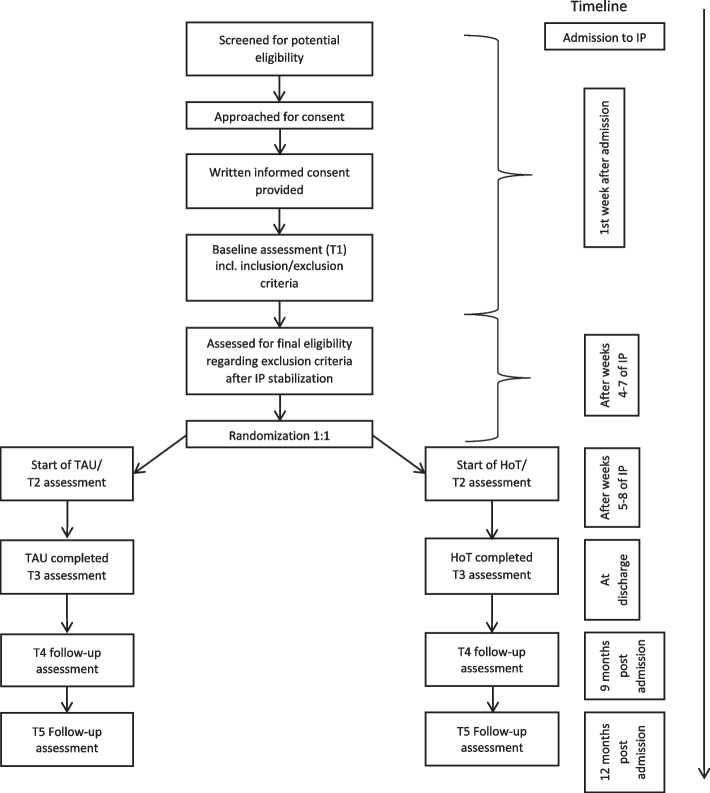
Diagram of patient flow in the RCT. Home treatment (HoT) versus treatment as usual (TAU). Abbr.: IP—inpatient treatment; incl.—including

**Fig. 2:**
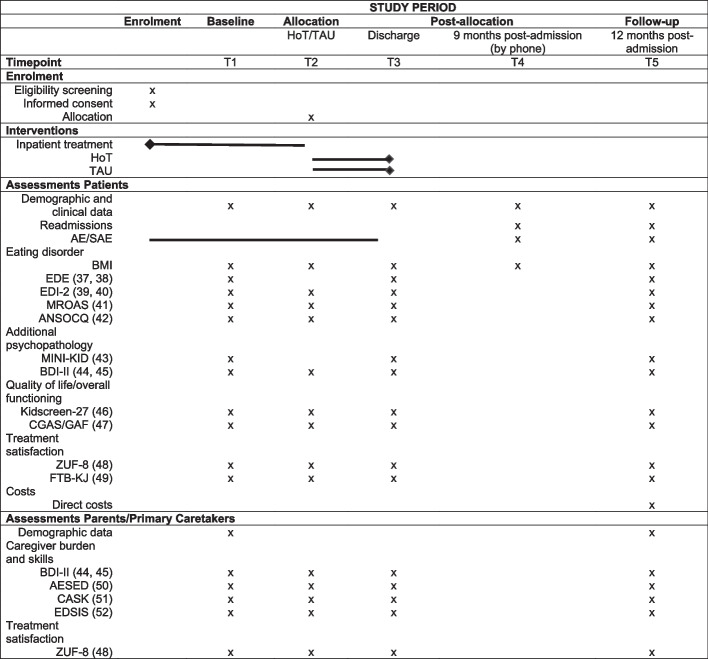
Schedule of enrollment, interventions, assessments, and measures of the RCT and patient data. Abbreviations: HoT – home treatment, TAU – treatment as usual, BMI – body mass index, EDE—Eating Disorder Examination Interview, EDI-2—Eating Disorder Inventory 2, MROAS—Morgan Russel Average Outcome Score, ANSOCQ—Anorexia Nervosa Stages of Change Questionnaire, MINI-KID—Mini-International Neuropsychiatric Interview for Children and Adolescents, BDI-II—Beck Depression Inventory 2, CGAS/GAF—Children’s Global Assessment Scale/Global Assessment of Functioning, ZUF—Satisfaction with treatment, FTB-KJ—Therapeutic Alliance Scale for Children, AE—adverse events, SAE—serious adverse events, AESED—Accommodation and Enabling Scale for Eating Disorders, CASK—Caregiver Skills, EDSIS—Eating Disorders Symptom Impact Scale [37–52]

In the RCT, standard care or treatment as usual (in this case IP/IP with DP via a stepped-care approach—TAU) is compared to the novel stepped-care treatment of HoT following short IP. Figure 1 shows a diagram of the patient flow through the RCT. Patients are screened for inclusion and exclusion criteria after hospital admission (Table 1 (a)). Patients are included in the trial after oral and written informed consent is provided by both the patients and their parents/legal guardians, and the families agreed to participate in the study (Fig. 1). Senior medical doctors or experienced psychologists will explain the details of the study and obtain informed consent. Three assessments take place during the intervention (T1, T2, and T3, Fig. 2), and two follow-up visits are made for the outcome assessment and to record adverse events (Figs. 1 and 2). After discharge from either treatment arm, the patients continue regular OP treatment as usually provided in Germany. This OP is not organized by the study sites and is not regulated by the study’s protocol; it does not necessarily need to take place at the study center and is not limited in duration, intensity, or type of therapy.

**Table 1:** Inclusion and exclusion criteria for home treatment

a. Criteria for hospital admission		
	Inclusion criteria	
		Diagnosis of AN or atypical AN according to the DSM-5
		Age ≥ 12 years and ≤ 18 years
		First or second inpatient admission because of AN
		Family lives within a 1-h commute of the treatment center
		Lives together with one or both parents (not residential treatment)
		Written informed assent of the patients and consent of the parents/legal guardians
	Exclusion criteria	
		Severe comorbid disorder (e.g., psychosis, bipolar disorder, substance dependence (except nicotine), severe self-harm)
		Current suicidal ideation
		Insufficient intellectual ability for a self-reliant home treatment (e.g., IQ ≤ 80)
		Severe comorbid somatic illness (e.g., diabetes mellitus type I)
		Insufficient knowledge of German
b. Criteria for randomization after 4 to 7 weeks of IP		
	Exclusion criteria	
		Persistent severe symptoms of AN (e.g., nasogastric tube feeding, independent eating at home is not possible)
		Severe psychological comorbidity (suicidality or severe self-harm)
		Severe somatic comorbidity
		Insufficient weight gain (less than 1.5 kg/4 weeks)
		Vomiting or laxative abuse > 2 times/day over > 1 week

Abbr.: *AN* anorexia nervosa; *DSM-5* Diagnostic and Statistical Manual of Mental Disorders, Fifth Edition; *IQ* Intelligence Quotient; *IP* inpatient treatment

### Participants and eligibility criteria

All adolescent patients who are diagnosed with AN or atypical AN (ICD-10: F 50.0 and F 50.1), who also meet the DSM-5 criteria, and who are consecutively admitted for IP treatment to the ED units at the sites, are screened for eligibility (Table 1 (a)). Inpatient admission is primarily carried out by the prescription of hospital treatment by general practitioners, pediatricians, or OP clinics due to the failure of OP treatment or by transfer from other hospitals. Parents/legal guardians as well as the patients are then approached by experienced psychologists or medical doctors and informed about all the details of the study. The inclusion process into the intervention is a two-stage procedure in which randomization into either treatment arm is conditional on further inclusion criteria of successful initial somatic stabilization (Table 1 (b)) based on the experiences of the pilot study [31] to avoid possible HoT-associated risks for the patients.

### Interventions

Treatment is provided in a multimodal, interdisciplinary setting. Psychotherapeutic interventions are provided according to the principles of CBT-E (Enhanced Cognitive Behavioral Therapy for Adolescents with Anorexia nervosa) [53], including family-based interventions (family sessions and family meals) encouraging parents to take the lead in refeeding their child [54]. The general treatment goals of AN-focused child and adolescent psychiatric and psychotherapeutic treatment in all participating departments during the intervention phases are depicted in Table 2. The same treatment approaches are provided during all phases of therapy, i.e., during IP before randomization and in both treatment arms (TAU and HoT) after randomization until discharge.

**Table 2:** AN treatment goals

General treatment goals	Specific treatment goals		
• weight rehabilitation • identification and reduction of eating disorder-specific cognitions and behaviors • normalization of eating behaviors • reduction of excessive exercise • improvement of distorted body image • guidance of parents/caregivers • reintegration into an age-appropriate everyday life • treatment of comorbid disorders	Inpatient stabilization (5–8 weeks)	HoT (12–16 weeks)	TAU (no length specified)
	• somatic stabilization • development of therapeutic relationship (patients/parents and staff) • psychoeducation (patients & parents) • instruction of parents to chaperone the meals and daily activities of the child (amount of food & extent of physical activity)	• treatment at home to achieve general treatment goals Special focus: • first 2 months: supporting parents as “co-therapists” (refeed child; support during food intake & ED-related behaviors) • third and fourth months: supporting patients in reintegration into an age-appropriate everyday life (e.g., regular school visits, social integration)	• continuation of IP/DP treatment to achieve general treatment goals • if possible, transfer to DP

Abbr.: *AN* anorexia nervosa, *HoT* home treatment, *TAU* treatment as usual, *ED* eating disorder, *DP* day patient treatment

### Inpatient treatment—somatic stabilization phase

During this phase (5–8 weeks), the patients are treated in the wards. All the patients undergo a multidisciplinary treatment program consisting of nutritional counseling and meal support, medical treatment, physiotherapy and occupational therapy, individual and group psychotherapy, parent and/or family sessions, and, if necessary, pharmacotherapy, according to the guidelines [8], see also Table 2. According to our experience the somatic complications of AN are usually stabilized during this time [31]. The target weight is determined, usually within the 20th–30th BMI percentile (approximately 90% EBW) on the basis of German population data, considering the individual weight/BMI levels prior to the onset of disease as well as the weight-associated increase in gonadotropine levels [26], especially in patients with atypical AN (AAN).

### Treatment as usual

The patients randomized into the TAU arm either stay in IP or are transferred to DP depending on individual needs or the usual treatment and situation at the respective center. TAU is continued for as long as it is individually necessary to achieve the target weight and sufficiently improve regarding cognitive, affective and behavioral symptoms of the ED (Table 2).

### HoT

**Fig. 3:**
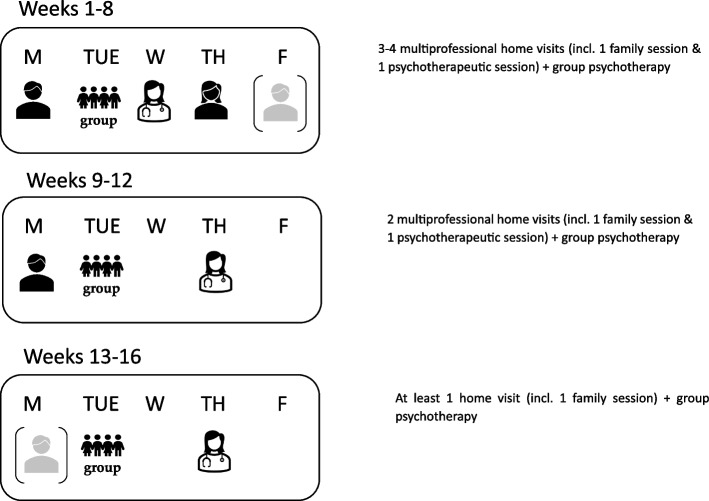
Appointment schedule of HoT. Abbr.: M—Monday; TUE—Tuesday; W—Wednesday; TH—Thursday; F—Friday; AN—Anorexia nervosa

Patients randomized into the HoT arm are discharged from the ward and HoT is carried out according to our manual for 12 to 16 weeks depending on individual needs. Psychological treatment is continued by the same therapists as during IP. Home treatment visits and clinic appointments are scheduled weekly in consultation with the family following the schedule depicted in Fig. 3. Patients receive continued multiprofessional treatment including medical care, at least weekly ED-focused cognitive-behavioral psychotherapy by a physician or a psychologist (s. above), nutritional therapy and family sessions, support by nursing staff, and other treatments such as occupational therapy and physiotherapy. One weekly session is planned as a family session involving at least one parent/primary caregiver and the patient. In addition to the home visits, patients also participate in a weekly AN-focused group psychotherapy session. During the first 2 months of HoT, parents/primary care-givers are supported in their role as “co-therapists”. Similar to FBT caregivers learn how to refeed their child, to help with food intake as well as with controlling excessive exercising or other disorder-related behaviors. In the 3rd and 4th months, the patient is supported in returning to a “normal life” including regular school visits, sports activities, and the re-uptake of hobbies and friendships.

### Criteria for discontinuing or modifying allocated interventions

When parents/patients discontinue treatment prematurely (e.g., discharge against medical advice), they are asked to still participate in the 9- and 12-month follow-up assessments to enable intention-to-treat analysis. Discontinuation of HoT may occur if one of the following criteria apply: (1) lack of compliance (if the patient or parents/legal guardians do not comply with the recommendations of the team (e.g., multiple appointment cancellations), (2) risks to patient safety (including serious medical complications, refusal to eat, suicidality, other serious adverse events that indicate a potential health hazard for the patient, insufficient weight gain, or other safety reasons which, in the opinion of the clinical team, preclude further treatment at home), or (3) the patient’s or parents’ wish to terminate home treatment (e.g., due to perceived burden on the family, increased conflicts, or other reasons). After discontinuing HoT, the patient is offered rehospitalization and treatment according to standard care. However, a short-term crisis leading to readmission of ≤ 7 days during HoT does not necessarily lead to a complete termination.

If patients or their legal guardians withdraw their consent for participation, HoT is discontinued and no further outcome assessments are performed, but standard treatment (e.g., as inpatients) is offered. For patients in the TAU arm, it is not possible to switch to HoT (as this is not a routinely offered treatment modality in Germany).

### Treatment completion

HoT and TAU are continued until the target weight is achieved and sustained, and cognitive, affective, and behavioral symptoms are alleviated. On average, a treatment period of 12 to 16 weeks for HoT was calculated. During the somatic stabilization phase and the treatment in either study arm (TAU, HoT), participants only receive treatment from the staff of the respective treatment site. Additional psychiatric and/or psychotherapeutic treatment is not allowed.

### Cost analysis

Direct costs for the health insurance institutions of the differential treatment period, i.e., between randomization (start of HoT/TAU) and discharge, will be collected and will be compared between the treatment arms.

### Implementation of HoT and staff survey

The feasibility and implementation of HoT in the individual departments are descriptively summarized. To identify the potential impact of HoT on staff, job satisfaction, stress, and workload of the multidisciplinary staff of HoT and the staff working at TAU are assessed. Four assessments are performed at each participating site throughout the trial (Table 3).

**Table 3:** Assessments and respective measures of the implementation and staff data

	**Before start of intervention**	**After 6 months of HoT at respective site**	**After 12 months of HoT at respective site**	**After 24 months of HoT at respective site**
**Timepoint**	M1	M2	M3	M4
**Assessments**				
UWES-9	x	x	x	x
BAUA-Scales	x	x	x	x
HoT-specific questionnaire of HoT staff only		x	x	x

Abbr.: *UWES-9* Utrecht Work Engagement Scale, *BAUA* Bundesanstalt für Arbeitsschutz und Arbeitsmedizin (Federal Institute of Safety at Work and Occupational Medicine)

### External validity and transferability

Since this trial is funded by German health insurance companies (“the Federal Joint Committee”), the potential transfer of HoT to the regular standard of care within the German health care system is of particular interest. An assessment of the transferability and external validity of the data will be carried out within this project using the data of the web-based German National Competency Network (“Kompetenznetz Anorexie-Register e. V.”), which contains the clinical data of children and adolescents, treated as inpatients at 16 mostly tertiary care hospitals specializing in ED treatment in Germany [55]. The HoT-data and the registry data will be compared regarding admission data to estimate the proportion of patients with AN in Germany who might be eligible for HoT. Careful interpretation will consider the non-randomized nature of such a comparison.

### Premature termination of the trial

The entire project may be terminated prematurely if patient inclusion is insufficient, or if the risks of the project outweigh the potential benefits, in that the safety and well-being of the participating patients cannot be guaranteed.

### Adaptations due to the COVID-19 pandemic

The study was planned prior to the COVID-19 pandemic. To meet restrictions (e.g., quarantines in the case of a COVID-19 infection), an adjustment had to be made to the protocol. Video consultations could replace home visits in the case of a COVID-19 infection for a maximum of 14 days.

### Strategies to improve adherence to interventions

#### Professional team

To monitor and improve adherence to the HoT intervention at the different treatment centers, all staff who work in the HoT arm received training from the coordinating physician of Aachen. In addition, each profession (e.g., nutritional therapist, nurse, etc.) was trained by the head of the respective field in Aachen. Additionally, to ensure adherence, at least yearly in-person meetings of the Aachen coordinating clinician are held with the treatment team of each site, in addition to regular biweekly supervision and exchange with the clinicians in charge at each center.

#### Families/patients

The treatment sessions are arranged with the family a week ahead of time. Patients or carers may receive additional appointments depending on their individual needs. In addition, to improve adherence, families and patients have the possibility of calling members of the treatment team during the day and the child and adolescent psychiatrist on call outside working hours. In the case of a crisis, a short-term hospitalization is possible at all sites to ensure patient and family adherence and safety.

### Outcomes

#### RCT

All outcomes of the RCT are assessed at five time points: (1) at hospital admission (T1), (2) at randomization (HoT/ TAU; T2), (3) at discharge (T3), (4) at 9 months after admission (interview by phone, T4), and (5) at 12 months after hospital admission (T5). Clinical interviews and examinations are conducted in person by experienced, trained, and (if necessary) certified medical doctors or psychologists, while self-report questionnaires are administered via the online tool “SoSci-Survey”, so that data entry and associated errors or missing values can be avoided. See Fig. 3 for a schematic diagram of the schedule of enrollment, interventions, assessments, and measures of the RCT and patient data.

##### Primary outcome

The primary outcome is the BMI (kg/m^2^) 12 months after hospital admission, which is assessed by staff blinded to the treatment allocation. The BMI is the most relevant indicator of long-term treatment success in adolescents with AN and has been used as the primary endpoint in many treatment studies [28, 56, 57]. BMI is calculated for all assessment time points from the measured height and weight of the patient in his or her underwear (except for self-reported values at T4, the interview via phone). Age- and sex-specific percentiles, SDS and %Expected Body Weight (%EBW) are calculated based on the large German reference dataset from the KIGGS study [58]. Recent studies have demonstrated that BMI alone might not be a meaningful prognostic indicator of a good long-term outcome in AAN [59]. Since randomization is stratified for BMI at admission (percentile < 10 vs. ≥ 10) and statistical analyses will be adjusted for BMI at admission and premorbid BMI, we still consider the increase in BMI to be a valid primary endpoint.

##### Secondary outcomes

Figure 3 shows all secondary outcomes (all at T5), e.g., ED symptoms and severity, other psychiatric comorbidities and quality of life, and all assessment instruments.

#### Cost analysis

Regarding the cost analysis, the direct costs of treatment (TAU vs. HoT between randomization and discharge) are collected from the centers.

#### Implementation and staff appraisal

For the descriptive implementation and feasibility analysis, treatment data are collected at each site: numbers of all AN patients screened, those who meet the inclusion criteria and are willing to participate in the study, the proportions of patients who were successfully stabilized and able to start and finish HoT according to the treatment plan, and the proportions of patients who do not meet the requirements for HoT, are analyzed. In addition, a descriptive evaluation of the actual weekly treatment implementation is carried out: the number of treatment sessions by the different occupational groups over the course of the HoT and the number of cancelled home visits. To assess the staff’s appraisal of HoT and the work satisfaction and stress of both groups (HoT/TAU), staff members of both treatment teams complete the short version of the Utrecht Work Engagement Scale, (UWES-9, [60]) and the “Bundesanstalt für Arbeitsschutz und Arbeitsmedizin” (Federal Institute of Safety at Work and Occupational Medicine)-Scales (BAUA-Scales, [61]). In addition to the validated questionnaires on work satisfaction and stress, a short debriefing is also designed for the therapists of the HoT team only. This questionnaire explicitly debriefs on stress and satisfaction with this specific new treatment. Table 3 displays the assessments of the staff.

#### Transferability

See above, 2.D.

##### Sample size and recruitment

The data of the HoT pilot study (*n* = 22) [31], the ANDI study (*n* = 172) [28], and the German Competence Network AN Register e.V. (*n* = 289) [55] were used as the basis for the statistical sample size planning of the RCT. In the IP arm, an increase in BMI of approximately 3 kg/m^2^ (SD 1.8) is expected after 1 year (ANDI study, [28]), and, in the HoT arm, an increase of 3.6 kg/m^2^ (SD 1.5; HoT pilot study) is expected. This translates to a standardized effect size of Cohen’s *d* = 0.47. For a statistical power of 80% (5% significance level), a sample size of 74 evaluable patients per study arm is needed. To account for approximately 10% loss-to-follow-up (missing values for the primary endpoint) [28], we plan to randomize 164 patients. Based on the experience of the pilot study, in which one third of the patients initially included at admission did not fulfill the criteria for transfer to HoT after 4 to 7 weeks of inpatient stabilization [31], a total of approximately 240 patients needs to be included in the study upon admission. To publicize the study, leaflets explaining it (reviewed and accepted by the respective ethics committees) are distributed regularly to general and pediatric practices as well as registered doctors and psychotherapists. Additionally, the consortium has its own website and email contacts and videos explaining the study. In addition, we give talks to local medical doctors and psychotherapists to explain the project.

## Methods: assignment of interventions

### Randomization and blinding

Randomization is carried out as block randomization using computer-generated random numbers, stratified by center, sex-, and age-specific BMI percentiles at admission (two strata: < 10th percentile vs. ≥ 10th percentile) at a ratio of 1:1 to the two treatment arms. Randomization lists were prepared by the Institute of Medical Informatics and Statistics at Kiel University (Prof. Dr. A. Dempfle). The randomization sequence is transferred to the participating treatment centers in sequentially numbered, sealed envelopes separately for each center-specific stratum (sex and age- and sex-specific BMI percentiles). Randomization in either the HoT arm of the study or the TAU arm takes place 1 week (± 2 days) before the start of the differential treatment, i.e., at the latest in the 7th week of IP. This time lag is necessary for organizational issues (e.g., parents taking time off from work, communicating with the school). If the patient meets the inclusion criteria for HoT after 4 to 7 weeks of inpatient treatment (and he or she included in the trial), the allocation is determined by having the approved staff at the treatment center open the respective envelope and communicate the news to the patient, family, and treatment team. Blinding of participants or study staff is not possible due to the nature of the treatments. However, a member of the staff who is assigned to another work area and who is not involved in any of the treatments assesses the clinical variables and conducts the interviews. Moreover, the BMI (weight in one’s underwear and height), an objective parameter, is used as the primary endpoint; a staff member who is blinded to the patient’s study arm (single-blind) assesses and records it.

## Methods: data collection, management, and analysis

### Data collection and management

The data management is carried out by the clinical trials center Kiel (ZKS Kiel) at Kiel University. Data collection at the respective centers is carried out with paper case report forms (CRFs) specifically prepared for this study in a pseudonymized manner in accordance with the ICH-GCP guidelines. Each included patient is assigned a numeric identification code to prevent identification. The master file containing the numeric codes and respective patient names will only be stored on paper, which is locked in a safe location with restricted access in the respective sites. This file will be deleted at the latest on May 31, 2035, 10 years after study completion, to fully anonymize the data. One copy of the CRF is sent to the clinical trials center (ZKS Kiel), where it is entered into an “Open Clinica” trial data base via double data entry. Data entry, safety, security, and storage are performed according to local standard operating procedures (SOPs). Standardization procedures, including methods to ensure standardization between sites, are developed and implemented across the sites to guarantee accurate, consistent, complete, and reliable data. In particular, the data collection and documentation are regularly monitored by a qualified monitor of the Clinical Trials Center Aachen (CTC Aachen) in accordance with the GCP guidelines and local SOPs.

### Statistical methods

#### RCT

The superiority of the HoT arm over the TAU arm will be analyzed for the primary endpoint (BMI 1 year after admission) using a linear mixed model for repeated measures in which study arm, sex, BMI at admission and at randomization, and age are included as fixed effects, and center and subject ID are included as random effects. All assessments of BMI after randomization (at discharge and at the 9- and 12-month follow-up timepoints) are considered repeated outcomes (the dependent variable); thus, time point is also included as a fixed effect. The difference between the achieved BMI in both study arms at the 12-month follow-up is the parameter of interest. Re-admissions are considered intercurrent events [62] and are handled depending on the timing of readmission. For patients who were readmitted to IP due to an eating disorder during follow-up and who were still in in- or day-patient treatment at the time of the 12-month follow-up assessment or discharged within the 4 weeks prior, we will use the BMI at readmission instead of that at follow-up to obtain a conservative estimate of their BMI. A subgroup analysis for patients with AAN (admission BMI ≥ 10th percentile) will be performed for the primary outcome. In addition, (serious) adverse events ((S)AEs) are tabulated by study arm and in relation to treatment as assessed by the investigator. No statistical tests will be performed for (S)AEs. Secondary endpoints are investigated with analogous logistic, linear or time-to-event (for time to readmission) models, depending on the type of endpoint.

The statistical evaluation of the RCT is carried out according to the intention-to-treat principle: all randomized study participants are included in the statistical analysis, regardless of whether the treatment was completed. In addition, an analysis only of patients treated in accordance with the protocol—i.e., without relevant protocol violations (per protocol set)—will be performed as a sensitivity analysis for the primary outcome. In general, the mixed model for repeated measures analysis can also be applied if some of the outcome data are missing. Further sensitivity analyses considering missing values will be performed.

#### Cost analysis

For the statistical evaluation of the cost data, cost from randomization to discharge will be compared between the study’s arms using a linear model with baseline covariates analogous to those for other quantitative outcome measures. Total costs from admission to discharge will also be reported for health insurance reasons.

#### Implementation process and staff evaluation

The implementation of HoT in the individual departments is analyzed descriptively: proportions of all AN patients who meet the inclusion criteria and/or who provide consent to participate in the study (including the reasons for rejection), the proportion of patients who are successfully stabilized and are allowed to start HoT and finish it regularly, and an analysis of patients who do not meet the requirements for HoT. The evaluation of the staff surveys regarding satisfaction and stress between HoT and TAU is analogous to the evaluation of the secondary endpoints of the RCT.

#### Assessment of transferability

The assessment of external validity and transferability is also carried out through a descriptive analysis (see 2.D above).

No interim analyses will be performed.

## Methods: oversight and monitoring

### Steering committee and coordinating center

A steering committee of the principal investigators at each clinical site and the health insurance companies is established and holds quarterly meetings to discuss and monitor issues that arise during the course of the study. Important protocol modifications are discussed within the steering committee and then distributed and communicated by the PIs of the respective centers. The team of the coordinating center consists of (1) a part-time senior psychologist in the role of the project coordinator who provides organizational support, supervises and supports the trial on a day-to-day basis, (2) a part-time coordinating physician, a senior medical doctor who trained all staff working in HoT at all sites, and is responsible for supervision and adherence on a day-to-day basis, (3) part-time senior psychologist/medical doctors who perform the assessments with included patients, and (4) the PI (medical consultant) who supervises and supports the whole trial. At every site, (1) a part-time project coordinator supervises the trial, and (2) a part-time senior psychologist performs the assessments. Meetings within and across sites take place weekly or biweekly.

### Data monitoring

This project and, in particular, the data collection and documentation, are regularly monitored by a qualified monitor of the Clinical Trials Center Aachen (CTC Aachen) in accordance with the GCP guidelines and local SOPs. At least four 2-day monitoring visits take place at each site over the study period. The monitoring is independent from the sponsor and the PIs. Besides the monitoring, which is independent from the sponsor and investigators, and the regular meetings within and across sites there are no more audits for trial conduct.

### Adverse event reporting and harms

In the pilot study with 22 patients, HoT was a safe treatment method. Except for deliberate self-harm which often occurs in adolescent patients presenting with an ED [63] no serious adverse events occurred [31]. In the present study, adverse events (AEs) or serious adverse events (SAEs) are documented, assessed, and reported according to GCP guidelines. SAEs are reported to the sponsor representative within 24 h. In the current multicenter trial, several precautions are taken to ensure the participant safety. First, the treatment centers were chosen based on their extensive and long-standing experience in treating adolescent patients with AN in IP and OP treatment settings. Second, each HoT staff member must have previous experience in treating adolescent patients with AN and must attend specific trainings. Additionally, close specialist supervision of the coordinating center and regular organizational meetings with clinical managers and senior psychiatrists is performed to address these challenges. In the case of a serious adverse event, an advisory board of two external experienced ED experts (Prof. Dr. Katrin Giel of Tübingen, Germany, and Prof. Dr. Martina De Zwaan of Hannover, Germany) review each case to recommend whether the trial should be continued and/or further measures of safety need to be implemented. The final decision in such cases lies with the PI as the sponsor representative. Insurance coverage for general harm exists through the liability insurance of the respective participating center. In addition, an insurance covering travel to and from the assessments at the respective centers has been established.

### Patient and public involvement

As part of the HoT pilot study [31], particular emphasis is given to assessing patients’ treatment satisfaction with HoT, the burden on caregivers, and the feasibility of such treatment for families. As a result, when planning this RCT, patients and parents who had lived experience were consulted on the organizational details of the HoT intervention and the trial to meet the wishes and requirements of the families and patients.

### Dissemination

The results and conclusions of this study will be presented at national and international conferences and will be published in peer-reviewed scientific journals. Additionally, the general results and conclusions will be disseminated on websites to ensure broad perceptions among the public. The findings will also be made available to the innovation fund at the Joint Federal Committee. If HoT is superior to TAU, we hope that home treatment is established as a treatment strategy in routine care for patients with AN, with treatment costs covered by statutory health insurance.

## Discussion

The outcome and chances of recovery for patients with AN have, if at all, improved only marginally in recent years. In her 2021 editorial, C. Bulik urgently called for new innovative, effective treatments for this vulnerable and burdened patient group [25], which has the highest mortality rate of all mental disorders [25]. She claims that our current postdischarge treatment—which has decreased from 24/7 treatment to one psychotherapy lesson per week—is not helpful for preventing relapse. Moreover, to help children or adolescents with AN, parents must be empowered and educated to become effective as co-therapists. Home treatment (HoT) with several weekly visits by a multidisciplinary team, seems to facilitate psychological, behavioral, and physical recovery “in the long run” because patients and carers are equally supported. Home treatment comprises some of the most effective therapeutic principles, including the direct environment, motivating the patient, and strengthening his or her autonomy. The current trial is the first RCT to investigate the effectiveness of a stepped-care approach of home treatment for children and adolescents with AN that is so severe that hospitalization seems inevitable. With this trial, we hope to demonstrate that a short inpatient stay followed by longer-term intensive support by an experienced team will be superior to a long inpatient stay. We will also investigate whether this new treatment strategy can be implemented in other departments for treating patients with eating disorders and whether the staff will appreciate and be able to manage this method. With this ambitious study plan in mind, we hope that this treatment approach will be successful in comparison to treatment as usual to inform future national and international treatment guidelines, thus providing hope to many patients and their carers.

### Limitations

One major limitation of our study is that the evaluation can only be partially blinded because the patients, the families and the therapists are aware of the respective treatment arm. Another limitation is that the time frames of the phases of inpatient treatment and home treatment are fixed so that the treatment phases cannot be individualized further.

## Conclusions

It has been already recognized for a considerable time that long hospitalizations can be detrimental to the healthy development of an adolescent. Moreover, several trials have successfully demonstrated that parents and family members involved in the care of patients should be included in the treatment process. Home treatment combines both advantages. If this approach proves feasible in various departments, for professionals as well as for the patients and their families, it might be possible to reduce the often early relapse rate in adolescent AN patients and thus improve the outcome of this often debilitating disorder.

## Trial status

Protocol version 5, 01.02.2023.

Participant recruitment and data collection started on November 30, 2021, and is expected to continue through April 2025.

Sponsor: GB-A Innovation fund, grant number 01VSF20006.

## Data Availability

The trial protocol, materials, and anonymized data will be made available by the Principal Investigator after having published major results and upon reasonable request.

## Abbreviations

AAN: Atypical anorexia nervosa
AESED: Accommodation and Enabling Scale for Eating Disorders
AN: Anorexia nervosa
ANSOCQ: Anorexia nervosa Stages of Change Questionnaire
BAUA: Bundesanstalt für Arbeitsschutz und Arbeitsmedizin
BDI-II: Beck Depression Inventory 2
BMI: Body mass index
CASK: Caregiver skills
CBT-E: Enhanced Cognitive Behavioral Therapy for Adolescents with Anorexia nervosa
CGAS/GAF: Children’s Global Assessment Scale/Global Assessment of Functioning
CRF: Case Report Form
CTC-A: Clinical Trials Center Aachen
DRKS: German Clinical Trials Register (Deutsches Register Klinischer Studien)
DP: Day patient treatment
DSM: Diagnostic and Statistical Manual of Mental Disorders
EBW: Expected body weight
ED: Eating disorder
EDE: Eating Disorder Examination Interview
EDI-2: Eating Disorder Inventory 2
EDSIS: Eating Disorders Symptom Impact Scale
FBT: Family-based treatment
FTB-KJ: Therapeutic Alliance Scale for Children
GCP: Good Clinical Practice
HoT: Home treatment
ICH: International Council for Harmonisation
IP: Inpatient treatment
MINI-KID: Mini-International Neuropsychiatric Interview for Children and Adolescents
MROAS: Morgan Russel Outcome Assessment Score
OP: Outpatient treatment
RCT: Randomized controlled trial
(S)AE: (Serious) adverse events
SD: Standard deviation
SDS: Standard deviation score
SOP: Standard Operating Procedure
TAU: Treatment as usual
UWES: Utrecht Work Engagement Scale
ZUF: Satisfaction with treatment Scale

## References

[1] WHO, Mental health: facing the challenges, building solutions: report from the WHO European MinisterialConference. Copenhagen: World Health Organization, Regional Office for Europe; 2005.

[2] Andres-Pepina S, Plana MT, Flamarique I, Romero S, Borras R, Julia L, et al. Long-term outcome and psychiatric comorbidity of adolescent-onset anorexia nervosa. Clin Child Psychol Psychiatry. 2020;25(1):33–44. 30764636 10.1177/1359104519827629

[3] Steinhausen HC, Villumsen MD, Horder K, Winkler LA, Bilenberg N, Stoving RK. Increased risk of somatic diseases following anorexia nervosa in a controlled nationwide cohort study. Int J Eat Disord. 2022;55(6):754–62. 35451527 10.1002/eat.23718 PMC9323483

[4] Auger N, Potter BJ, Ukah UV, Low N, Israel M, Steiger H, et al. Anorexia nervosa and the long-term risk of mortality in women. World Psychiatry. 2021;20(3):448–9. 34505367 10.1002/wps.20904 PMC8429328

[5] Burns J, Shank C, Ganigara M, Saldanha N, Dhar A. Cardiac complications of malnutrition in adolescent patients: a narrative review of contemporary literature. Ann Pediatr Cardiol. 2021;14(4):501–6. 35527750 10.4103/apc.apc_258_20 PMC9075577

[6] Solmi M, Monaco F, Hojlund M, Monteleone AM, Trott M, Firth J, et al. Outcomes in people with eating disorders: a transdiagnostic and disorder-specific systematic review, meta-analysis and multivariable meta-regression analysis. World Psychiatry. 2024;23(1):124–38. 38214616 10.1002/wps.21182 PMC10785991

[7] Austin A, Flynn M, Richards K, Hodsoll J, Duarte TA, Robinson P, et al. Duration of untreated eating disorder and relationship to outcomes: a systematic review of the literature. Eur Eat Disord Rev. 2021;29(3):329–45. 32578311 10.1002/erv.2745

[8] AWMF S3-Leitlinie Diagnostik und Therapie der Essstörungen. 2018. https://register.awmf.org/de/leitlinien/detail/051-026. Accessed 10 July 2024.

[9] NICE – National Institute for Health and Care Excellence. Eating disorders: recognition and treatment. London: National Institute for Health and Care Excellence guideline (NG69); 2017.

[10] Lock J. An update on evidence-based psychosocial treatments for eating disorders in children and adolescents. J Clin Child Adolesc Psychol. 2015;44(5):707–21. 25580937 10.1080/15374416.2014.971458

[11] Jaite C, Buhren K, Dahmen B, Dempfle A, Becker K, Correll CU, et al. Clinical characteristics of inpatients with childhood vs. adolescent anorexia nervosa. Nutrients. 2019;11(11):2593. 31661861 10.3390/nu11112593 PMC6893829

[12] Quadflieg N, Naab S, Schlegl S, Bauman T, Voderholzer U. Inpatient treatment outcome in a large sample of adolescents with anorexia nervosa. Nutrients. 2023;15(19):4247. 37836531 10.3390/nu15194247 PMC10574756

[13] Fennig S, Brunstein Klomek A, Shahar B, Sarel-Michnik Z, Hadas A. Inpatient treatment has no impact on the core thoughts and perceptions in adolescents with anorexia nervosa. Early Interv Psychiatry. 2017;11(3):200–7. 25808049 10.1111/eip.12234

[14] Treasure J, Claudino AM, Zucker N. Eating disorders. Lancet (London, England). 2010;375(9714):583–93. 19931176 10.1016/S0140-6736(09)61748-7

[15] Hemmingsen SD, Jensen NA, Larsen PV, Sjogren JM, Lichtenstein MB, Stoving RK. Cortisol, depression, and anxiety levels before and after short-term intensive nutritional stabilization in patients with severe anorexia nervosa. Front Psychiatry. 2022;13: 939225. 35903636 10.3389/fpsyt.2022.939225 PMC9314772

[16] Walsh BT, Xu T, Wang Y, Attia E, Kaplan AS. Time course of relapse following acute treatment for anorexia nervosa. Am J Psychiatry. 2021;178(9):848–53. 34154394 10.1176/appi.ajp.2021.21010026 PMC8440387

[17] Castro J, Gila A, Puig J, Rodriguez S, Toro J. Predictors of rehospitalization after total weight recovery in adolescents with anorexia nervosa. Int J Eat Disord. 2004;36(1):22–30. 15185268 10.1002/eat.20009

[18] Meule A, Schrambke D, Furst Loredo A, Schlegl S, Naab S, Voderholzer U. Inpatient treatment of anorexia nervosa in adolescents: a 1-year follow-up study. Eur Eat Disord Rev. 2021;29(2):165–77. 33230832 10.1002/erv.2808

[19] Herpertz-Dahlmann B, Dempfle A. Treatment setting matters - An evaluation of prognostic factors for outcome in adolescent AN. NY, USA: Proceedings of the 22nd Meeting of the Eating Disorder Research Society, New York; 2016.

[20] Herrmann K, Kaluscha R, Liebert A, Spohrs J, Gundel H, von Wietersheim J. First onset of treatment of patients with eating disorders and treatment course: Results of data from a German health insurance company. Eur Eat Disord Rev. 2022;30(6):787–96. 35590442 10.1002/erv.2922

[21] Hay PJ, Touyz S, Claudino AM, Lujic S, Smith CA, Madden S. Inpatient versus outpatient care, partial hospitalisation and waiting list for people with eating disorders. Cochrane Database Syst Reviews. 2019;1:CD010827. 10.1002/14651858.CD010827.pub2 PMC6353082 30663033

[22] Kaye WH, Bulik CM. Treatment of patients with anorexia nervosa in the US-A Crisis in Care. JAMA Psychiat. 2021;78(6):591–2. 10.1001/jamapsychiatry.2020.4796 33625500

[23] Haripersad YV, Kannegiesser-Bailey M, Morton K, Skeldon S, Shipton N, Edwards K, et al. Outbreak of anorexia nervosa admissions during the COVID-19 pandemic. Arch Dis Child. 2021;106(3): e15. 32709684 10.1136/archdischild-2020-319868

[24] Herpertz-Dahlmann B, Dempfle A, Eckardt S. The youngest are hit hardest: the influence of the COVID-19 pandemic on the hospitalization rate for children, adolescents, and young adults with anorexia nervosa in a large German representative sample. Eur Psychiatry. 2022;65(1): e84. 36403977 10.1192/j.eurpsy.2022.2345 PMC9748980

[25] Bulik CM. From awareness to action: an urgent call to address the inadequacy of treatment for anorexia nervosa. Am J Psychiatry. 2021;178(9):786–8. 34516232 10.1176/appi.ajp.2021.21070697

[26] Herpertz-Dahlmann B. Intensive treatments in adolescent anorexia nervosa. Nutrients. 2021;13(4):1265. 33924294 10.3390/nu13041265 PMC8068891

[27] Madden S, Miskovic-Wheatley J, Wallis A, Kohn M, Lock J, Le Grange D, et al. A randomized controlled trial of in-patient treatment for anorexia nervosa in medically unstable adolescents. Psychol Med. 2015;45(2):415–27. 25017941 10.1017/S0033291714001573 PMC4301212

[28] Herpertz-Dahlmann B, Schwarte R, Krei M, Egberts K, Warnke A, Wewetzer C, et al. Day-patient treatment after short inpatient care versus continued inpatient treatment in adolescents with anorexia nervosa (ANDI): a multicentre, randomised, open-label, non-inferiority trial. Lancet. 2014;383(9924):1222–9. 24439238 10.1016/S0140-6736(13)62411-3

[29] Treasure J, Willmott D, Ambwani S, Cardi V, Clark Bryan D, Rowlands K, Schmidt U. Cognitive interpersonal model for anorexia nervosa revisited: the perpetuating factors that contribute to the development of the severe and enduring illness. J Clin Med. 2020;9(3):630. 32120847 10.3390/jcm9030630 PMC7141127

[30] Davis L, Walsh BT, Schebendach J, Glasofer DR, Steinglass JE. Habits are stronger with longer duration of illness and greater severity in anorexia nervosa. Int J Eat Disord. 2020;53(5):413–9. 32227516 10.1002/eat.23265 PMC7217727

[31] Herpertz-Dahlmann B, Borzikowsky C, Altdorf S, Heider K, Dempfle A, Dahmen B. 'Therapists in action'-Home treatment in adolescent anorexia nervosa: A stepped care approach to shorten inpatient treatment. European eating disorders review : the journal of the Eating Disorders Association. 2020. 10.1002/erv.2755 32558214

[32] Flutsch N, Hilti N, Schraer C, Soumana M, Probst F, Haberling I, et al. Feasibility and acceptability of home treatment as an add-on to family based therapy for adolescents with anorexia nervosa. A case series. Int J Eating Disord. 2021;54(9):1707–10. 10.1002/eat.23567 PMC8457051 34227130

[33] Pauli D, Flutsch N, Hilti N, Schraer C, Soumana M, Haberling I, Berger G. Home treatment as an add-on to family-based treatment in adolescents with anorexia nervosa: A pilot study. Eur Eating Disord Rev : J Eating Disord Association. 2022;30(2):168–77. 10.1002/erv.2882 PMC9303788 35001459

[34] Heider KS, Dempfle A, Altdorf S, Herpertz-Dahlmann B, Dahmen B. Motivation to change in the course of a pilot study of a step-down treatment approach of inpatient and anorexia nervosa-specific home treatment and its effects on treatment outcome. Front Psychiatry. 2021;12:693103. 34690825 10.3389/fpsyt.2021.693103 PMC8529001

[35] Altdorf S, Dempfle A, Heider K, Seitz J, Herpertz-Dahlmann B, Dahmen B. [Parents as co-therapists in home treatment for adolescents with anorexia nervosa - factors and mechanisms]. Prax Kinderpsychol Kinderpsychiatr. 2022;71(5):467–86. 35786438 10.13109/prkk.2022.71.5.467

[36] Chan AW, Tetzlaff JM, Altman DG, Dickersin K, Moher D. SPIRIT 2013: new guidance for content of clinical trial protocols. Lancet (London, England). 2013;381(9861):91–2. 23305999 10.1016/S0140-6736(12)62160-6

[37] Fairburn CG, Cooper Z. The eating disorder examination. In: Fairburn CG, Wilson GT, editors. Binge eating: nature, assessment, and treatment. 12th ed. New York: Guilford Press; 1993. p. 317–56.

[38] Hilbert A, Tuschen-Caffier B. Eating disorder examination: Deutschsprachige Übersetzung. Münster: Verlag für Psychotherapie; 2006.

[39] Garner DM. Eating disorder inventory-2: professional manual. Odessa: Psychological Assessment Resources; 1991.

[40] Paul T, Thiel, A. Eating Disorder Inventory - 2: EDI-2; Deutsche Version, Manual. Göttingen: Hogrefe; 2005.

[41] Morgan HG, Hayward AE. Clinical assessment of anorexia nervosa. The Morgan-Russell outcome assessment schedule. Br J Psychiatry. 1988;152:367–71. 10.1192/bjp.152.3.367 3167372

[42] Rieger E, Touyz SW, Beumont PJ. The Anorexia Nervosa Stages of Change Questionnaire (ANSOCQ): information regarding its psychometric properties. Int J Eat Disord. 2002;32(1):24–38. 12183943 10.1002/eat.10056

[43] Sheehan DV, Sheehan KH, Shytle RD, Janavs J, Bannon Y, Rogers JE, et al. Reliability and validity of the Mini International Neuropsychiatric Interview for Children and Adolescents (MINI-KID). J Clin Psychiatry. 2010;71(3):313–26. 20331933 10.4088/JCP.09m05305whi

[44] Hautzinger M, Keller F, Kühner C. BDI-II: beck-depressions-inventar. In: GmbH PAI, editor. 2006.

[45] Hautzinger M, Keller F, Kühner C. Beck Depressions Inventar, 2. Auflage (BDI-II). Göttingen: Hogrefe; 2006.

[46] Ravens-Sieberer U, Auquier P, Erhart M, Gosch A, Rajmil L, Bruil J, et al. The KIDSCREEN-27 quality of life measure for children and adolescents: psychometric results from a cross-cultural survey in 13 European countries. Qual Life Res. 2007;16(8):1347–56. 17668292 10.1007/s11136-007-9240-2

[47] Shaffer D, Gould MS, Brasic J, Ambrosini P, Fisher P, Bird H, Aluwahlia S. A children’s global assessment scale (CGAS). Arch Gen Psychiatry. 1983Nov 1;40(11):1228–31. 6639293 10.1001/archpsyc.1983.01790100074010

[48] Schmidt J, Lamprecht F, Wittmann WW. [Satisfaction with inpatient management. Development of a questionnaire and initial validity studies]. Psychotherapie, Psychosomatik, medizinische Psychologie. 1989;39(7):248–55. 2762479

[49] Kronmüller KT, Hartmann M, Reck C, Victor D, Horn H, Winkelmann K. Die therapeutische Beziehung in der Kinder-und Jugendlichen-Psychotherapie. Z Klin Psychol Psychother. 2003;32(1):14–23.

[50] Sepulveda AR, Kyriacou O, Treasure J. Development and validation of the accommodation and enabling scale for eating disorders (AESED) for caregivers in eating disorders. BMC Health Serv Res. 2009;9: 171. 19775448 10.1186/1472-6963-9-171 PMC2759929

[51] Hibbs R, Rhind C, Salerno L, Lo Coco G, Goddard E, Schmidt U, et al. Development and validation of a scale to measure caregiver skills in eating disorders. Int J Eat Disord. 2015;48(3):290–7. 25351932 10.1002/eat.22362

[52] Sepulveda AR, Whitney J, Hankins M, Treasure J. Development and validation of an Eating Disorders Symptom Impact Scale (EDSIS) for carers of people with eating disorders. Health Qual Life Outcomes. 2008;6: 28. 18426597 10.1186/1477-7525-6-28 PMC2365933

[53] Dalle Grave R, Conti M, Calugi S. Effectiveness of intensive cognitive behavioral therapy in adolescents and adults with anorexia nervosa. Int J Eat Disord. 2020;53(9):1428–38. 32691431 10.1002/eat.23337

[54] Forsberg S, Lock J. Family-based Treatment of Child and Adolescent Eating Disorders. Child Adolesc Psychiatr Clin N Am. 2015;24(3):617–29. 26092743 10.1016/j.chc.2015.02.012

[55] Buhren K, Herpertz-Dahlmann B, Dempfle A, Becker K, Egberts KM, Ehrlich S, et al. First Sociodemographic, Pretreatment and Clinical Data from a German Web-Based Registry for Child and Adolescent Anorexia Nervosa. Zeitschrift fur Kinder- und Jugendpsychiatrie und Psychotherapie. 2017;45(5):393–400. 28825513 10.1024/1422-4917/a000544

[56] Lock J, Agras WS, Le Grange D, Couturier J, Safer D, Bryson SW. Do end of treatment assessments predict outcome at follow-up in eating disorders? Int J Eat Disord. 2013;46(8):771–8. 23946139 10.1002/eat.22175

[57] Glasofer DR, Muratore AF, Attia E, Wu P, Wang Y, Minkoff H, et al. Predictors of illness course and health maintenance following inpatient treatment among patients with anorexia nervosa. J Eat Disord. 2020;8(1):69. 33292619 10.1186/s40337-020-00348-7 PMC7709230

[58] Schaffrath Rosario A, Kurth BM, Stolzenberg H, Ellert U, Neuhauser H. Body mass index percentiles for children and adolescents in Germany based on a nationally representative sample (KiGGS 2003–2006). Eur J Clin Nutr. 2010;64(4):341–9. 20179728 10.1038/ejcn.2010.8

[59] Walsh BT, Hagan KE, Lockwood C. A systematic review comparing atypical anorexia nervosa and anorexia nervosa. Int J Eat Disord. 2023;56(4):798–820. 36508318 10.1002/eat.23856

[60] Sautier LP, Scherwath A, Weis J, Sarkar S, Bosbach M, Schendel M, et al. Assessment of work engagement in patients with hematological malignancies: psychometric properties of the German Version of the Utrecht Work Engagement Scale 9 (UWES-9). Rehabilitation (Stuttg). 2015;54(5):297–303. 26505182 10.1055/s-0035-1555912

[61] Nübling M, Stößel U, Hasselhorn HM, Michaelis M, Hofmann F. Methoden zur Erfassung psychischer Belastungen. In: Erprobung eines Messinstrumentes (COPSOQ). Wirtschaftsverlag NW; 2005.

[62] Nübling M, Stößel U, Hasselhorn H-M, Michaelis M, Hofmann F. Methoden zur Erfassung psychischerBelastungen. Erprobung eines Messinstrumentes (COPSOQ). Dortmund: Wirtschaftsverlag NW; 2005.

[63] European Medicines Agency. ICH E9 (R1) addendum on estimands and sensitivity analysis in clinical trials to the guideline on statistical principles for clinical trials. 2020. https://www.ema.europa.eu/en/documents/scientificguideline/ich-e9-r1-addendum-estimands-and-sensitivity-analysis-clinical-trials-guideline-statistical-principlesclinical-trials-step-5_en.pdf. Accessed on 9 May 2024.

